# Considerations in the choice of side in a free Latissimus Dorsi flap to determine expendability in extensive lower extremity defects

**DOI:** 10.4103/0970-0358.63941

**Published:** 2010

**Authors:** Sunderraj Ellur, S. P. Bharath

**Affiliations:** St John's Medical College and Hospital, Bangalore, India

Sir,

The Latissimus Dorsi is a useful free flap for coverage of extensive defects of the lower limb. In such cases, despite successful microvascular coverage, the patient may still be crutch dependent. In such circumstances, the Latissimus Dorsi is an important muscle for crutch walking. "Do not burn your bridges" is an important rule in reconstructive surgery. We have reviewed the literature regarding shoulder weakness after Latissimus Dorsi harvest and presented a simple rule regarding choice of side when this flap is used for lower extremity defects. We also present a case where we had an opportunity to apply this rule.

**Figure 1:**
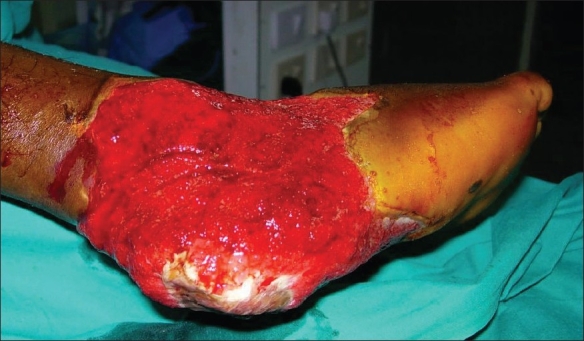
Left heel defect

**Figure 2:**
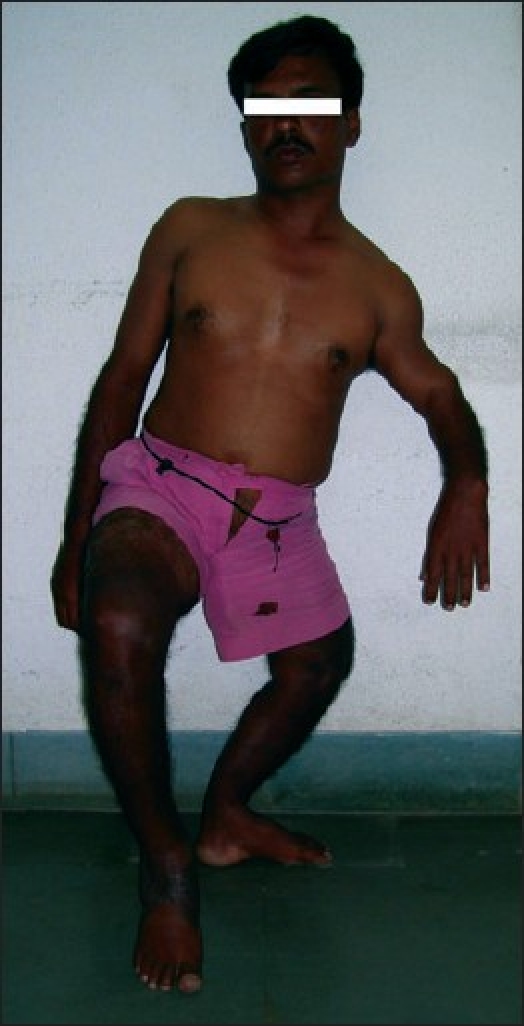
Patient profile showing short and deformed left lower limb

**Figure 3:**
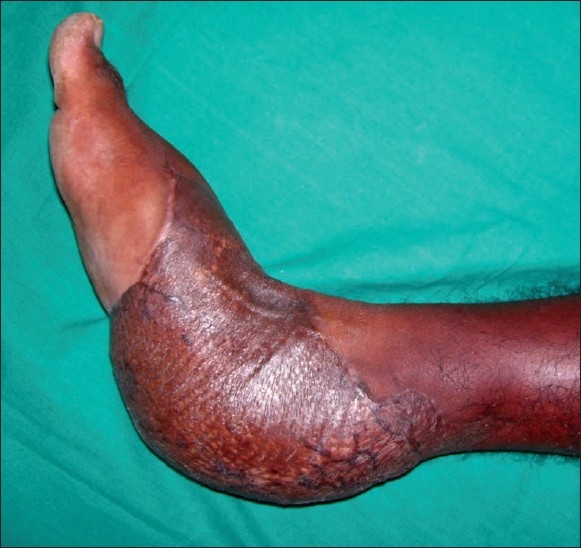
Healed Latissimus Dorsi flap - medial view

**Figure 4:**
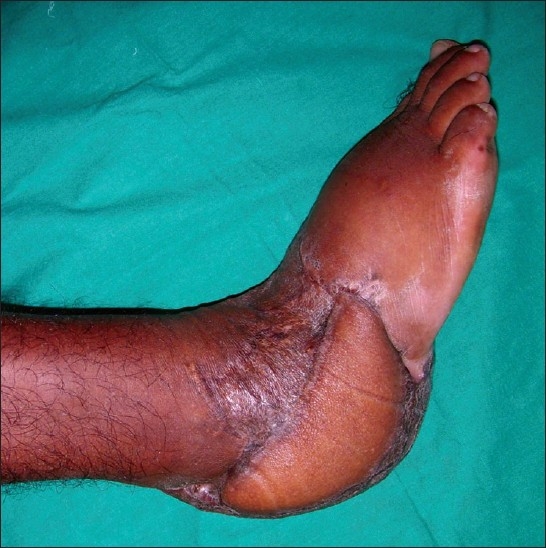
Healed Latissimus Dorsi flap - lateral view

A male patient aged 28 years presented to us with a history of road traffic accident resulting in extensive degloving injury of the right heel with exposure of the calcaneum [Figure 1]. We planned a free Latissimus Dorsi flap for wound cover. This patient also had congenital deformity of his left upper and lower limbs, with significant shortening of the left lower limb [Figures 2]. He was using a crutch for ambulation since his childhood. In view of the congenital shortening deformity in the left lower limb, we preferred to harvest the right Latissimus Dorsi muscle for free flap transfer because he would need the left Latissimus Dorsi muscle for crutch walking. After 1 year follow-up, the patient did not complain of any weakness during crutch walking [Figures 3 and 4.

Latissimus Dorsi is a Latin term, meaning "widest of the back". The Latissimus is one of the 26 muscles that make up the shoulder joint complex.[1] The Latissimus muscle acts on the humerus in adduction, medial rotation, extension and downward rotation of the scapula. This action is possible through the synergistic actions of the Latissimus with six other muscles[2] (pectoralis major, subscapularis, deltoid, teres major, teres minor and coracobrachialis, of which the teres major muscle is the principle component).

The Latissimus Dorsi free flap is well established as the workhorse in extensive defects of the lower limb. Although one article, as early as 1955, warned against the use of Latissimus Dorsi in certain circumstances like poliomyelitis,[3] in which it may be the "only lateral muscle capable of elevating the pelvis for a forward step", major text books describe the functional loss of this muscle as minimal. This conclusion, however, is based on few objective studies. There have been several studies which have shown, objectively, varying degrees of weakness in shoulder function after a free Latissimus Dorsi muscle transfer.

Laitung and Peck found a higher disability score after recent surgery than after longer time. Overall, 47% of the patients reported subjective difficulty in performing activities of daily living (most commonly lifting).[4] Russel *et al*. described a loss of strength of 9.1% for shoulder adduction and 10.5% for shoulder extension after the loss of the Latissimus Dorsi muscle. The authors also concluded that synergistic muscle groups assume much of the Latissimus function with time. Using the American Medical Association Guides to Evaluation of Permanent Impairment, they found that the shoulder disability averaged 6.2% for their entire series.[5] Brumback *et al*. found that approximately one-third of the patients who participate in sporting activities, including swimming, may be unable to do so satisfactorily after Latissimus Dorsi transfer.[6] Fraulin *et al*. demonstrated, in dynamic muscle tests, a deficit of muscle power and endurance of shoulder extension and adduction following Latissimus Dorsi muscle transfer.[7] Ishida *et al*. studied the relationship between age, time and strength after Latissimus Dorsi transfer and concluded that the older the patient was, greater was the weakness in the shoulder and this weakness reduced with time after the Latissimus Dorsi transfer as the synergic muscle activity seemed to compensate the missing Latissimus Dorsi.[8] Adams William *et al*. found that the work performance for Latissimus Dorsi muscle activities like ladder climbing and pushing up from a chair for the operated side had mean scores of 77–84% of the nonoperated site.[9]

From the above review of the literature, it appears that a careful history and examination of the patients with details of hobbies and athletic pursuits is essential to assess expendability.

Traditionally, the considerations in choice of a particular side, for a Latissimus Dorsi flap transfer were:

1. Simultaneous two-team approach (without a need to change position)
2. Donor vessel
3. Previous axillary dissection
4. Shape of the defect
5. Shape of the skin paddle needed
6. Any previous trauma to the back

We propose, that in microvascular Latissimus reconstructions for extensive lower limb defects, the opposite Latissimus Dorsi muscle be chosen for reconstruction. In extensive lower extremity defects, there is always a possibility of limb amputation if the microvascular reconstruction fails. In such a situation, the ipsilateral Latissimus Dorsi muscle will be invaluable for crutch walking. Further, if a patient is crutch dependent despite a successful microvascular cover, then the patient would need the ipsilateral Latissimus muscle. Another way of addressing this problem is to consider a segmental Latissimus dorsi transfer with functional preservation of the reminder muscle.

In conclusion, the possibility of weakness in shoulder movements must be taken into account when choosing this muscle as a donor. While planning Latissimus Dorsi muscle free flap for extensive lower extremity defects, one must choose the contralateral muscle to spare the ipsilateral muscle in anticipation of crutch walking. Most surgeons look at immediate considerations such as patient position and operating time; however, we must look beyond, at the patient's long-term needs, while choosing the flap side.
